# Supplementary material for: Functional characterisation of Target of Rapamycin (TOR) signalling in Physcomitrella
Source: Plant Cell Rep. 2026 Jan 30;45(2):46. doi: 10.1007/s00299-026-03722-y (PMC12858634; doi:10.1007/s00299-026-03722-y)
Supplement: Supplementary file 1 — Supplementary file1 Supplementary Figure S1 Expression levels of TORC1 component genes in Physcomitrella at different culture conditions and developmental stages. Supplementary Figure S2 Caulonema frequency increased with incubation time. Supplementary Figure S3 Phylogeny of the plant RAPTOR family. Supplementary Figure S4 Complementation of the yeast tor and raptor mutants with their Physcomitrella homologs. Supplementary Figure S5 Gametophore counts over time in Physcomitrella WT. Supplementary Figure S6 Expression levels of PpS6K genes in Physcomitrella at different culture conditions and developmental stages. Supplementary Figure S7 Expression levels of PpRPS6 genes in Physcomitrella at different culture conditions and developmental stages. Supplementary Figure S8 Yeast complementation assay of the fkbp12Δ mutant with mutant FKBP12 variants from Physcomitrella and Arabidopsis. Supplementary Table S1 Primers used to clone ScFKBP12, PpFKBP12 WT and mutants in p415 GAL1 restricted with XbaI and XhoI. Supplementary Table S2 Primers used to generate the vectors containing the different PIG1bL-CaMV 35Sp-FKBP12-cMyc-CaMV 35St-PIG1bR DNA constructs, to validate via PCR their correct integration at the PIG1 locus, and to determine via qPCR their copy number in the Physcomitrella genome. Supplementary Table S3 Primers used to generate the estradiol-inducible lst8, tor, and raptors RNA interference (RNAi) systems destined for a targeted integration at the PIG1 locus in the Physcomitrella genome. Supplementary Table S4 International Moss Stock Center (IMSC, www.mossstock-center.org) accession numbers of the lines used in this study. (DOCX 6375 KB) [file 299_2026_3722_MOESM1_ESM.docx]

**Supplementary information**

**
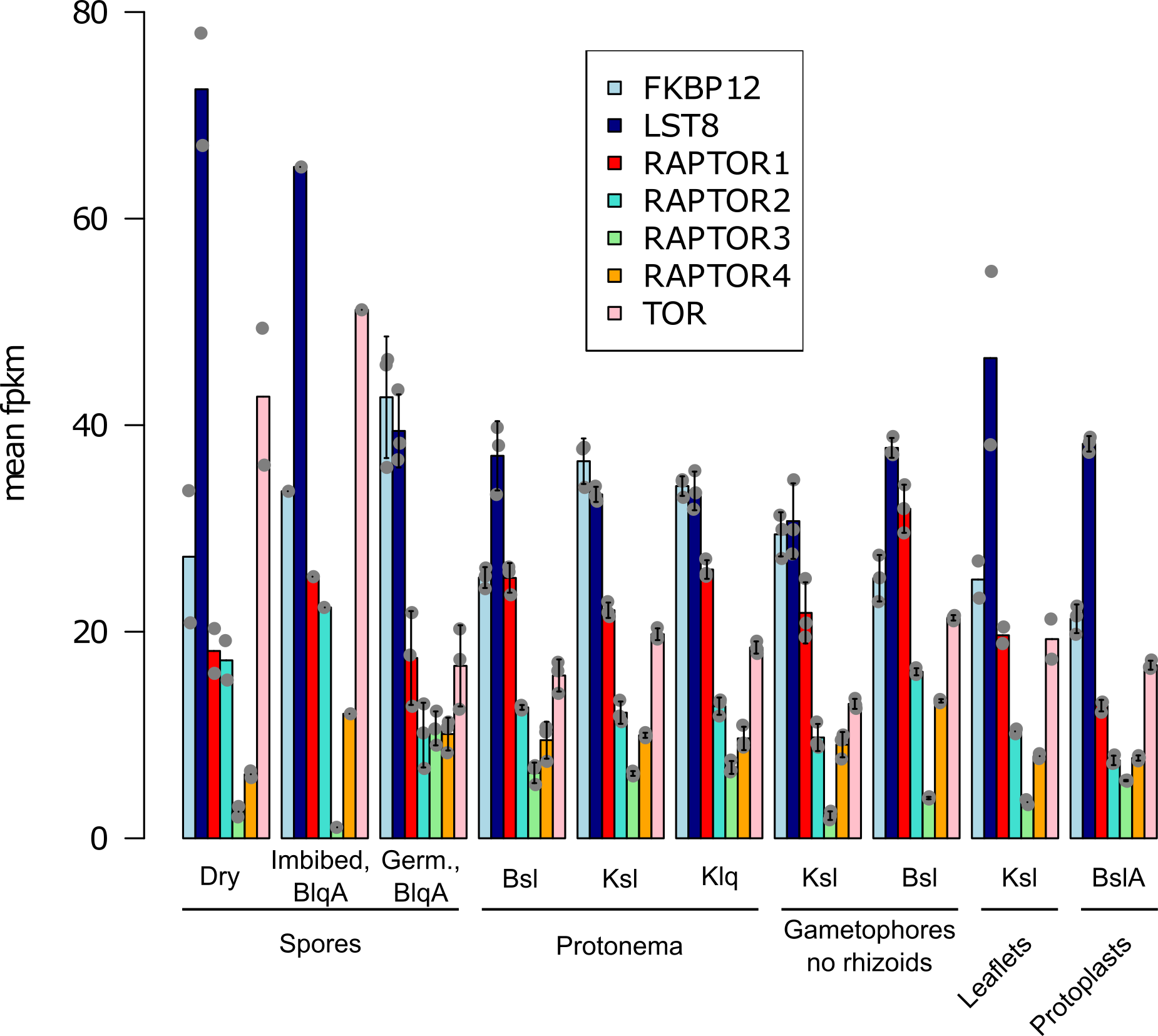
**

**Supplementary Figure S1 Expression levels of TORC1 component genes in Physcomitrella at different culture conditions and developmental stages.**

Expression data were retrieved from *PEATmoss* (https://peatmoss.plantcode.cup.uni-freiburg.de/expression_viewer/input). Values represent mean FPKM values (Fragments Per Kilobase Million) and standard deviation. Data points represent biological replicates. Abbreviations: B = BCD medium; lq = liquid; A = ammonium tartrate; sl = solid; K = Knop medium; Germ. = germinating.


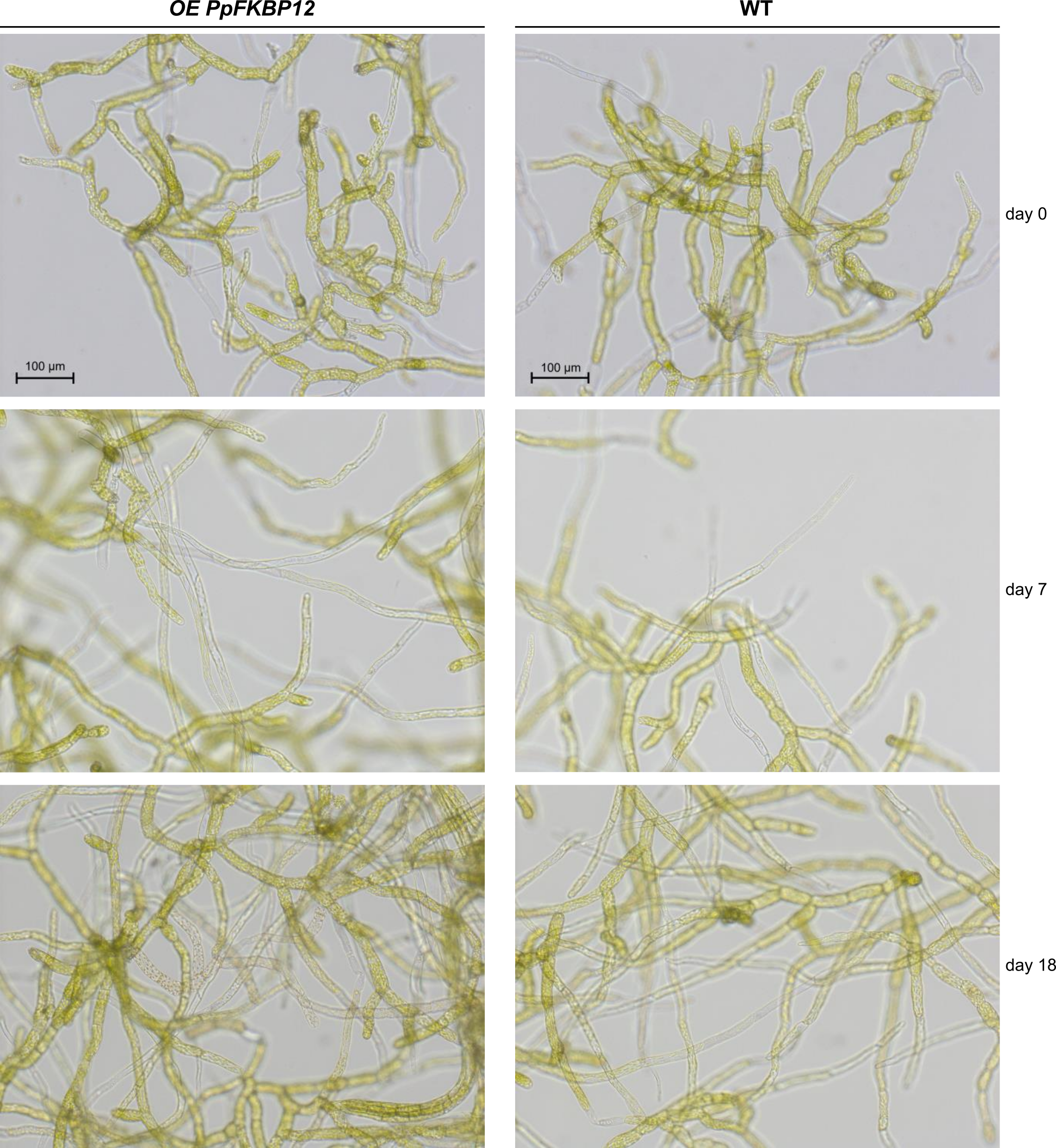


**Supplementary Figure S2 Caulonema frequency increased with incubation time.**

Light microscopy images of protonemata in suspension cultures treated with DMSO 0.01 % and incubated for 7 and 18 days. Culture conditions are similar to those in Figure 1.


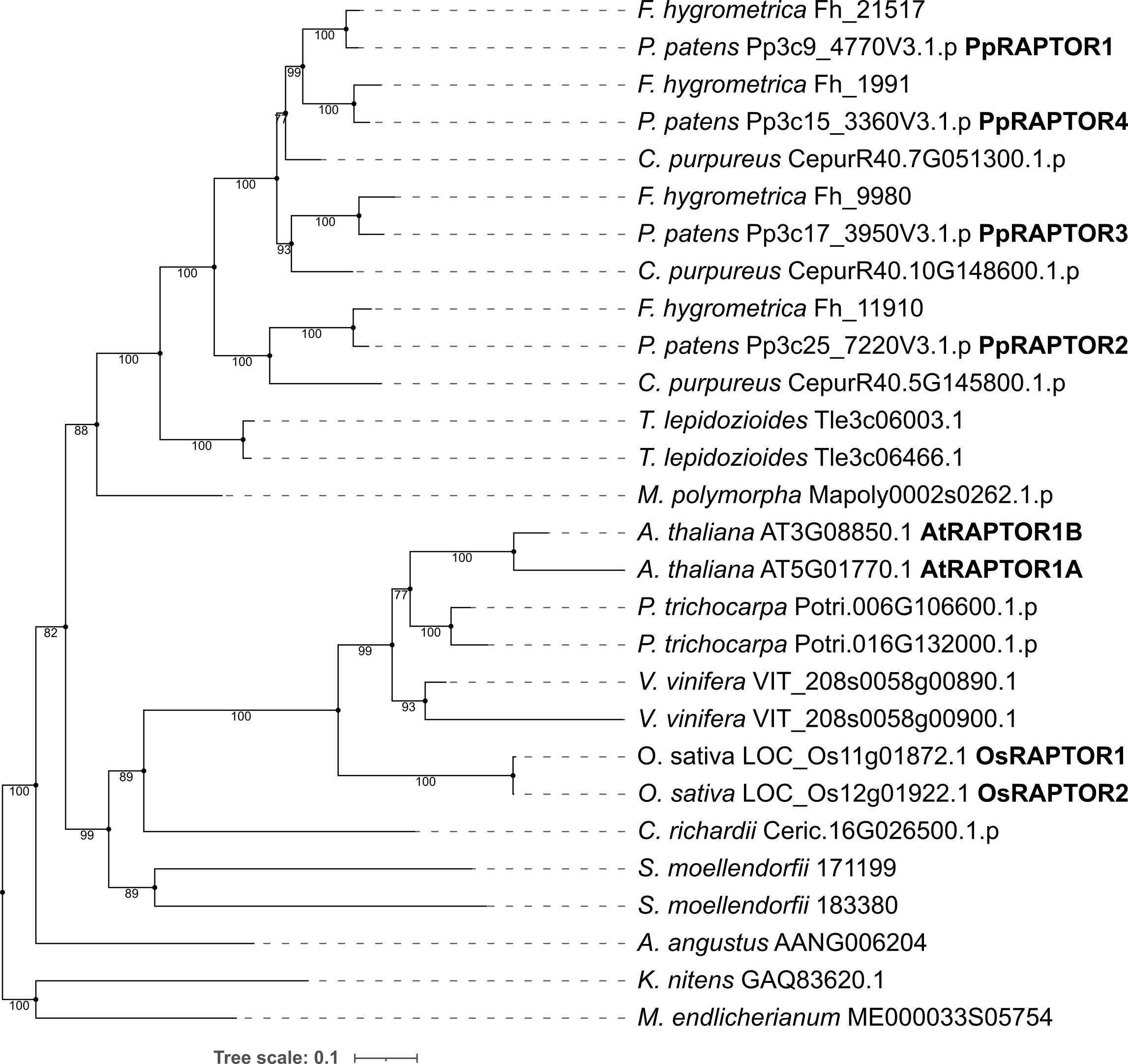


**Supplementary Figure S3 Phylogeny of the plant RAPTOR family.**

Maximum-likelihood tree based on a multiple sequence alignment of RAPTOR proteins, rooted at the split between streptophytic algae and land plants. Common names of RAPTOR isoforms are written in bold where available. Numeric values at nodes indicate percentage support based on 1000 bootstrap trees.


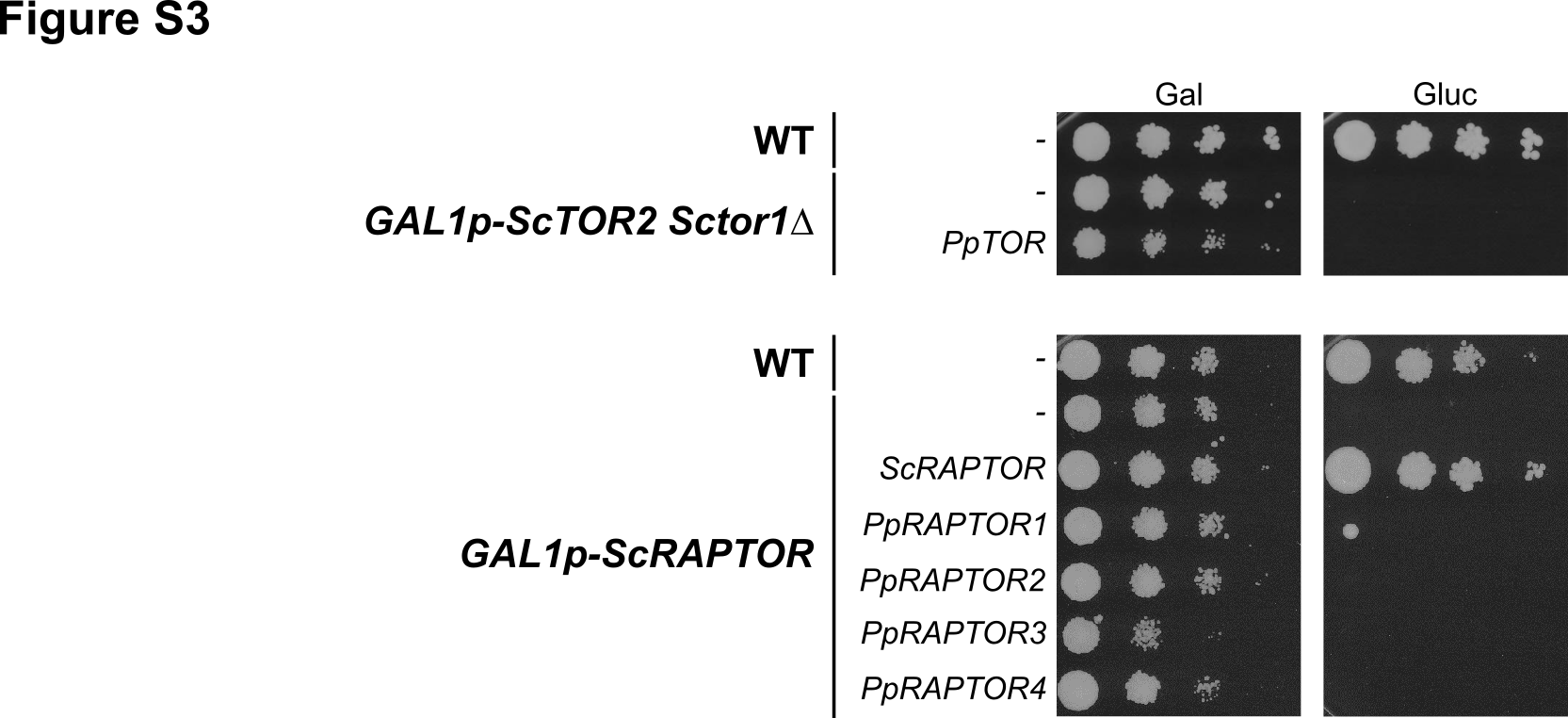


**Supplementary Figure S4 Complementation of the yeast *tor* and *raptor* mutants with their Physcomitrella homologs.**

**A** Wild-type TB50a yeast strain and mutant SW137-4b expressing *ScTOR2* from the galactose-inducible GAL1 promoter were initially grown as suspensions in rich medium containing galactose as a carbon source (YPGal). Strains were transformed with pESPB019 (empty vector) or pESPB022 (pESPB019-derived vector expressing *PpTOR*) and selected on SGal-Leu plates. Selected transformants were grown for 6 h at 30°C in SD-Leu suspension cultures. Cultures were normalized to an OD (660 nm) of 0.2, subjected to 10-fold serial dilutions, and 15 µl of each dilution were spotted onto SGal-Leu or SD-Leu plates, which were incubated at 30°C for 5 days. Plates were scanned upside down with the lid open on a Canon CanoScan 9000F scanner. **B** Same growth conditions as in panel A. Strain RL93a expressing yeast *RAPTOR* from the GAL1 promoter was transformed with pESPB019 (empty vector), pESPB019-derived vectors each containing one of the Physcomitrella RAPTOR paralogs (pESPB024, pESPB026, pESPB029, and pESPB032), or pCHD001 expressing *ScRAPTOR*.

**
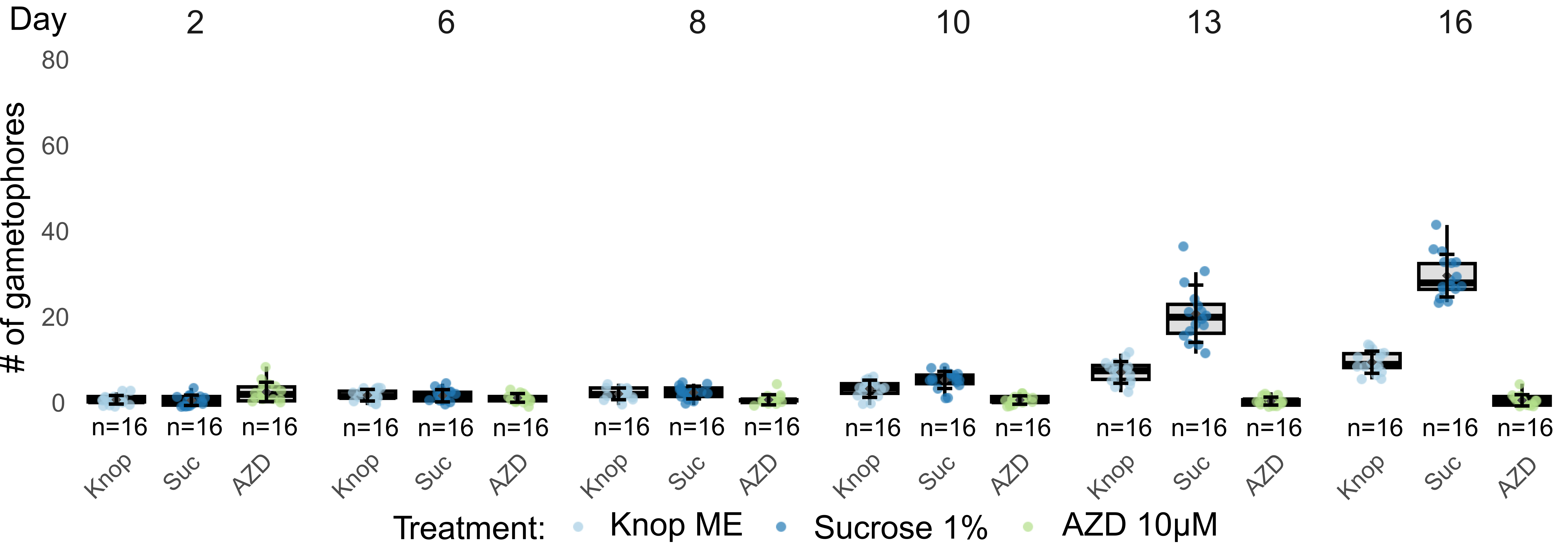
**

**Supplementary Figure S5 Gametophore counts over time in Physcomitrella WT.** Protonema at a density of 440 mg DW/L was spotted on solid medium plates (15 µL) containing either only Knop ME or additionally 1% sucrose or 10 µM AZD8055. Arising gametophores were counted over 16 days. Each data point represents a single spot at which gametophores were counted. n = 16 colonies per treatment.

**
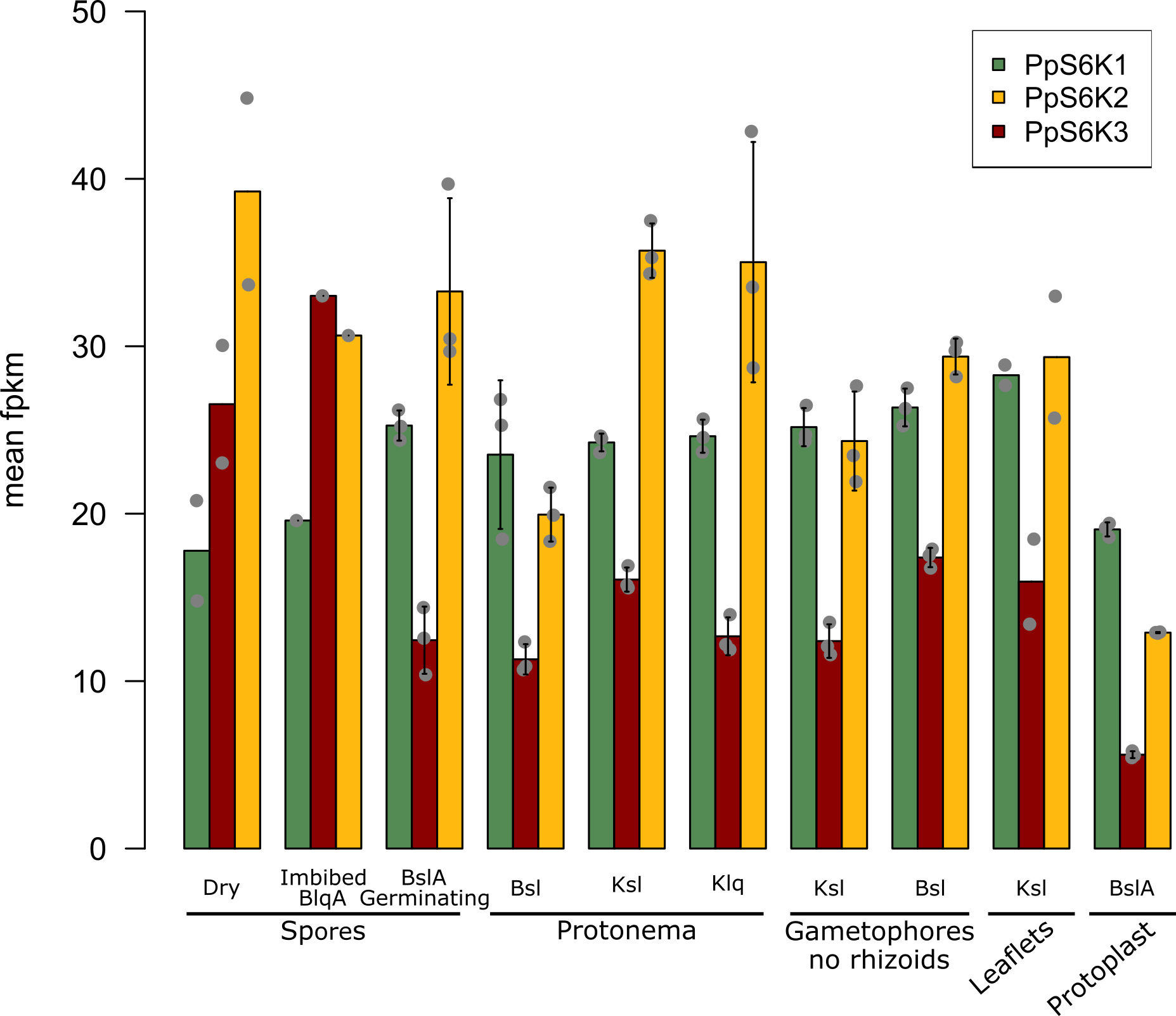
**

**Supplementary Figure S6 Expression levels of *PpS6K* genes in Physcomitrella at different culture conditions and developmental stages.**

Expression data were retrieved from *PEATmoss* (https://peatmoss.plantcode.cup.uni-freiburg.de/expression_viewer/input). Values represent mean FPKM values (Fragments Per Kilobase Million) and standard deviation. Data points represent biological replicates. Abbreviations: B = BCD medium; lq = liquid; A = ammonium tartrate; sl = solid; K = Knop medium; Germ. = germinating. PpS6K1: Pp3c12_223 60V3.1; PpS6K2: Pp3c25_14820V3.1; PpS6K3: Pp3c16_24840V3.1

**
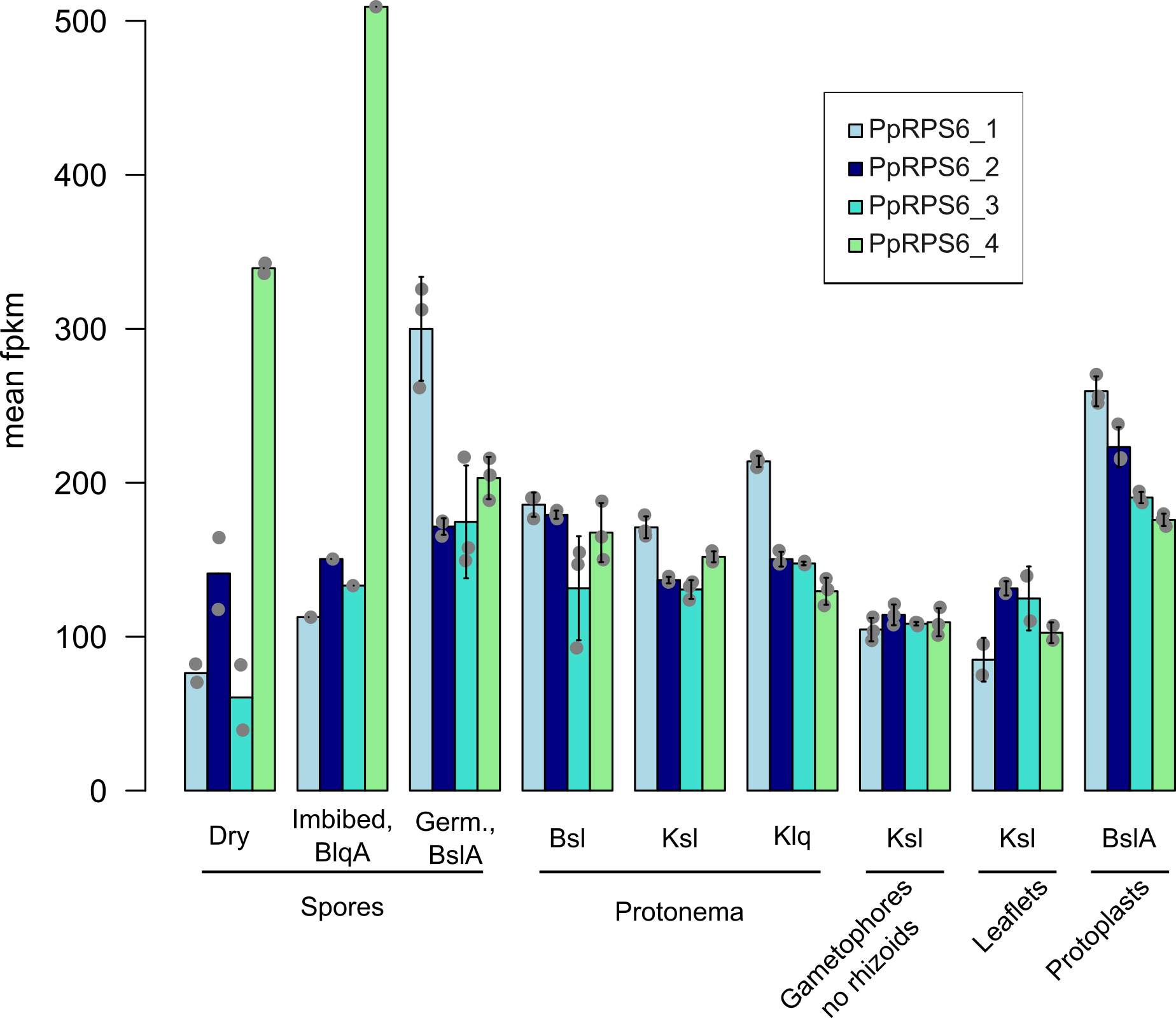
**

**Supplementary Figure S7 Expression levels of *PpRPS6* genes in Physcomitrella at different culture conditions and developmental stages.**

Expression data were retrieved from *PEATmoss* (https://peatmoss.plantcode.cup.uni-freiburg.de/expression_viewer/input). Values represent mean FPKM values (Fragments Per Kilobase Million) and standard deviation. Data points represent biological replicates. Abbreviations: B = BCD medium; lq = liquid; A = ammonium tartrate; sl = solid; K = Knop medium; Germ. = germinating. PpRPS6_1: Pp3c11_21120V3.1; PpRPS6_2: Pp3c15_25430V3.1; PpRPS6_3: Pp3c15_25780V3.1; PpRPS6_4: Pp3c7_5780V3.1


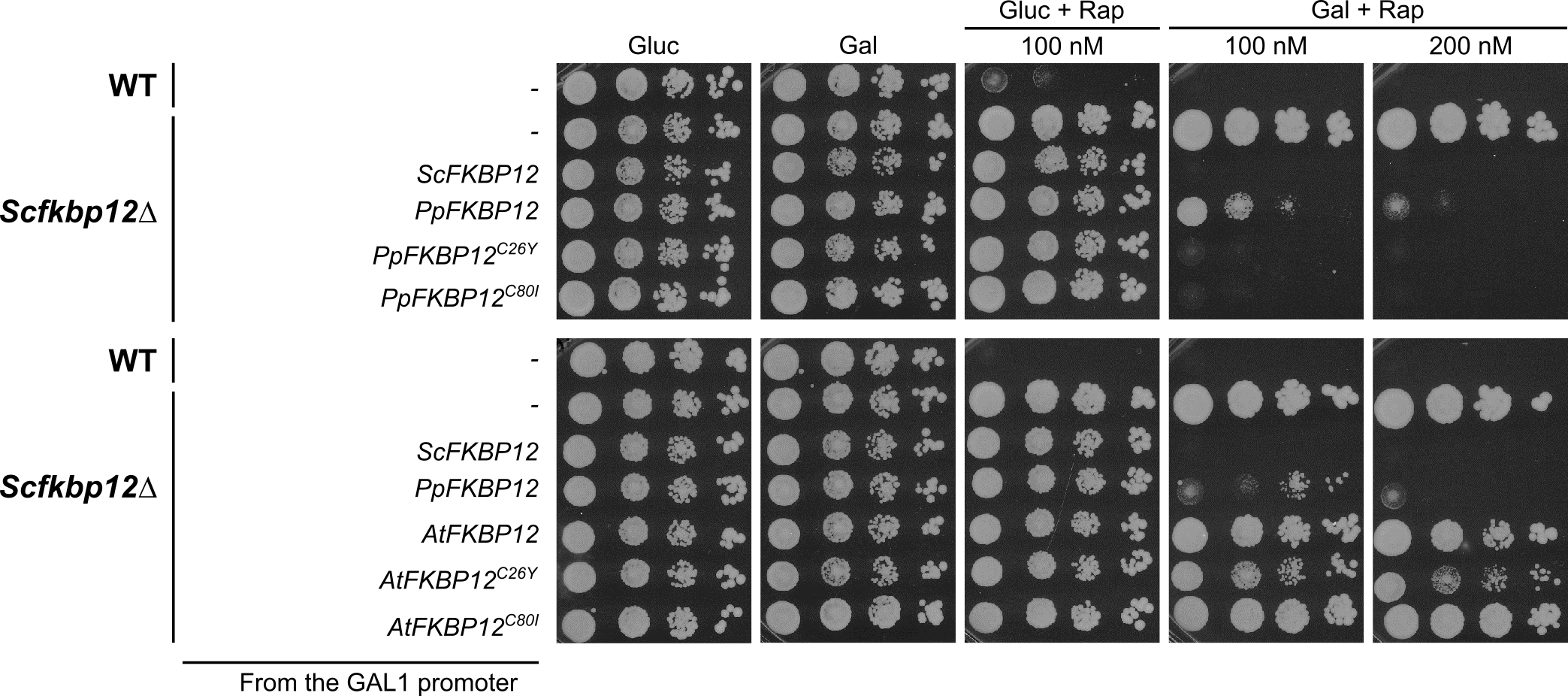


**Supplementary Figure S8 Yeast complementation assay of the *fkbp12Δ* mutant with mutant FKBP12 variants from Physcomitrella and Arabidopsis.**

Wild-type MH272-3c yeast strain transformed with an empty vector (p415 GAL1), and mutant MH274-1a (*fkbp12Δ*) strain transformed with either p415 GAL1 or p415 GAL1-derived vectors expressing different *FKBP12* variants, were grown in SD-Leu or SGal-Leu suspension cultures. Cultures were normalized to an optical density (OD) at 660 nm of 0.1, subjected to 10-fold serial dilutions, and 15 µL of each dilution were spotted onto SD-Leu or SGal-Leu plates supplemented with DMSO (0.02%) or with rapamycin at 100 or 200 nM. Plates were incubated at 30°C for 5 days.

**Supplementary Table S1**

**Primers used to clone *ScFKBP12*, *PpFKBP12* WT and mutants in p415 GAL1 restricted with XbaI and XhoI.** Grey font colours indicate that the primers have been re-used.

| **Plasmid** | **DNA template** | **Primer name** | **Primer sequence** |
| --- | --- | --- | --- |
| pESPB001 | Yeast gDNA | 5_Cl_ScFKBP12 | ACGTCAAGGAGAAAAAACCCCGGATTCTAGAatgtctgaagtaattgaaggtaac |
|  |  | 3_Cl_ScFKBP12 | AAGCGTGACATAACTAATTACATGACTCGAGttagttgaccttcaacaattcgac |
| pESPB003 | Physcomitrella cDNA | 5_Cl_PpFKBP12wt | ACGTCAAGGAGAAAAAACCCCGGATTCTAGAatggggtacgagaaagaaatc |
|  |  | 3_Cl_PpFKBP12wt | AAGCGTGACATAACTAATTACATGACTCGAGtcactgggcgctgagc |
| pESPB006 | pESPB003 | 5_Cl_PpFKBP12wt | ACGTCAAGGAGAAAAAACCCCGGATTCTAGAatggggtacgagaaagaaatc |
|  |  | 3_Cl_57_PpFKBP12C26Y | GAACTTCTCATTCAGGTCCCTATTCTTGCCATAGCCGGTGTagtggacagtgacggtttg |
|  |  | 5_Cl_57_PpFKBP12C26Y | CAAACCGTCACTGTCCACTAcaccggctatggcaagaatagggacctgaatgagaagttc |
|  |  | 3_Cl_PpFKBP12wt | AAGCGTGACATAACTAATTACATGACTCGAGtcactgggcgctgagc |
| pESPB008 | pESPB003 | 5_Cl_PpFKBP12wt | ACGTCAAGGAGAAAAAACCCCGGATTCTAGAatggggtacgagaaagaaatc |
|  |  | 3_Cl_77_PpFKBP12W40D | GCCAGGGTCCTTTGTACTATCgaacttctcattcaggtccctattcttgccatagccggtg |
|  |  | 5_Cl_77_PpFKBP12W40D | CACCGGCTATGGCAAGAATAGGGACCTGAATGAGAAGTTCGATagtacaaaggaccctggc |
|  |  | 3_Cl_PpFKBP12wt | AAGCGTGACATAACTAATTACATGACTCGAGtcactgggcgctgagc |
| pESPB010 | pESPB003 | 5_Cl_PpFKBP12wt | ACGTCAAGGAGAAAAAACCCCGGATTCTAGAatggggtacgagaaagaaatc |
|  |  | 3_Cl_57_PpFKBP12C26Y | GAACTTCTCATTCAGGTCCCTATTCTTGCCATAGCCGGTGTagtggacagtgacggtttg |
|  |  | 5_Cl_77_PpFKBP12W40D | CACCGGCTATGGCAAGAATAGGGACCTGAATGAGAAGTTCGATagtacaaaggaccctggc |
|  |  | 3_Cl_PpFKBP12wt | AAGCGTGACATAACTAATTACATGACTCGAGtcactgggcgctgagc |
| pESPB012 | pESPB003 | 5_Cl_PpFKBP12wt | ACGTCAAGGAGAAAAAACCCCGGATTCTAGAatggggtacgagaaagaaatc |
|  |  | 3Cl_146_PpFKBP12M56V-R58Q | CATCCCAACCTTTGATCACTTGACCCACccctaactcaaactggaag |
|  |  | 5Cl_146_PpFKBP12M56V-R58Q | CTTCCAGTTTGAGTTAGGGGTGGGTCAagtgatcaaaggttgggatg |
|  |  | 3_Cl_PpFKBP12wt | AAGCGTGACATAACTAATTACATGACTCGAGtcactgggcgctgagc |
| pESPB014 | pESPB003 | 5_Cl_PpFKBP12wt | ACGTCAAGGAGAAAAAACCCCGGATTCTAGAatggggtacgagaaagaaatc |
|  |  | 3Cl_146_PpFKBP12- M56K-G57Q-R58E | CATCCCAACCTTTGATCACTTCCTGCTTccctaactcaaactggaag |
|  |  | 5Cl_146_PpFKBP12- M56K-G57Q-R58E | CTTCCAGTTTGAGTTAGGGAAGCAGGAAgtgatcaaaggttgggatg |
|  |  | 3_Cl_PpFKBP12wt | AAGCGTGACATAACTAATTACATGACTCGAGtcactgggcgctgagc |
| pESPB016 | pESPB010 | 5_Cl_PpFKBP12wt | ACGTCAAGGAGAAAAAACCCCGGATTCTAGAatggggtacgagaaagaaatc |
|  |  | 3Cl_146_PpFKBP12M56V-R58Q | CATCCCAACCTTTGATCACTTGACCCACccctaactcaaactggaag |
|  |  | 5Cl_146_PpFKBP12M56V-R58Q | CTTCCAGTTTGAGTTAGGGGTGGGTCAagtgatcaaaggttgggatg |
|  |  | 3_Cl_PpFKBP12wt | AAGCGTGACATAACTAATTACATGACTCGAGtcactgggcgctgagc |
| pESPB018 | pESPB010 | 5_Cl_PpFKBP12wt | ACGTCAAGGAGAAAAAACCCCGGATTCTAGAatggggtacgagaaagaaatc |
|  |  | 3Cl_146_PpFKBP12- M56K-G57Q-R58E | CATCCCAACCTTTGATCACTTCCTGCTTccctaactcaaactggaag |
|  |  | 5Cl_146_PpFKBP12- M56K-G57Q-R58E | CTTCCAGTTTGAGTTAGGGAAGCAGGAAgtgatcaaaggttgggatg |
|  |  | 3_Cl_PpFKBP12wt | AAGCGTGACATAACTAATTACATGACTCGAGtcactgggcgctgagc |

**Supplementary Table S2**

**Primers used to generate the vectors containing the different *PIG1bL-CaMV 35Sp-FKBP12-cMyc-CaMV 35St-PIG1bR* DNA constructs, to validate via PCR their correct integration at the PIG1 locus, and to determine via qPCR their copy number in the Physcomitrella genome.** Grey font colours indicate that the primers have been re-used.

| **Purpose** | **Obtained vector name** | **DNA template used for PCR** | **Primer name** | **Primer sequence** |
| --- | --- | --- | --- | --- |
| DNA construct for the targeted integration of *PpFKBP12* fused to *cMyc* | pESPB033 | Bel5-PIG1b-Act5 | 3Cl_5HR-PIG1 | TGTATTCTATTTGATTGATAAGAAAAATGATAATAGTTTAAAGAGC |
|  |  |  | 5Cl_5HR-PIG1 | AAAACATGAATAACCAAATTAAAATATTAATAATTCATAGGG |
|  |  | mAV4-hpt | 5Cl_5HR-PIG1_35S-P | GCTCTTTAAACTATTATCATTTTTCTTATCAATCAAATAGAATACAGCGGCCGCctgcaggtccccagattagc |
|  |  |  | 3Cl_35S-P | TCTAGAtgttctctccaaatgaaatgaacttcc |
|  |  | 2027_pluc-nosp_35st | 5Cl_Myc_35S-T | GAACAAAAACTCATCTCAGAAGAGGATCTGTGACTCGAGgtccgcaaaaatcaccagtc |
|  |  |  | 3Cl_5HR-PIG1_35S-T | CCCTATGAATTATTAATATTTTAATTTGGTTATTCATGTTTTGCGGCCGCgtcactggattttggttttagg |
|  |  | pESPB003 | 5Cl_35S-P_PpFKBP12 | GGAAGTTCATTTCATTTGGAGAGAACATCTAGAatggggtacgagaaagaaatcatccggcc |
|  |  |  | 3Cl_Myc_PpFKBP12 | CAGATCCTCTTCTGAGATGAGTTTTTGTTCctgggcgctgagcacctcgatttc |
| DNA construct for the targeted integration of *PpFKBP12-C26Y* fused to *cMyc* | pESPB035 | Bel5-PIG1b-Act5 | 3Cl_5HR-PIG1 | TGTATTCTATTTGATTGATAAGAAAAATGATAATAGTTTAAAGAGC |
|  |  |  | 5Cl_5HR-PIG1 | AAAACATGAATAACCAAATTAAAATATTAATAATTCATAGGG |
|  |  | mAV4-hpt | 5Cl_5HR-PIG1_35S-P | GCTCTTTAAACTATTATCATTTTTCTTATCAATCAAATAGAATACAGCGGCCGCctgcaggtccccagattagc |
|  |  |  | 3Cl_35S-P | TCTAGAtgttctctccaaatgaaatgaacttcc |
|  |  | 2027_pluc-nosp_35st | 5Cl_Myc_35S-T | GAACAAAAACTCATCTCAGAAGAGGATCTGTGACTCGAGgtccgcaaaaatcaccagtc |
|  |  |  | 3Cl_5HR-PIG1_35S-T | CCCTATGAATTATTAATATTTTAATTTGGTTATTCATGTTTTGCGGCCGCgtcactggattttggttttagg |
|  |  | pESPB006 | 5Cl_35S-P_PpFKBP12 | GGAAGTTCATTTCATTTGGAGAGAACATCTAGAatggggtacgagaaagaaatcatccggcc |
|  |  |  | 3Cl_Myc_PpFKBP12 | CAGATCCTCTTCTGAGATGAGTTTTTGTTCctgggcgctgagcacctcgatttc |
| DNA construct for the targeted integration of *PpFKBP12-C26Y* fused to *cMyc* | pESPB037 | Bel5-PIG1b-Act5 | 3Cl_5HR-PIG1 | TGTATTCTATTTGATTGATAAGAAAAATGATAATAGTTTAAAGAGC |
|  |  |  | 5Cl_5HR-PIG1 | AAAACATGAATAACCAAATTAAAATATTAATAATTCATAGGG |
|  |  | mAV4-hpt | 5Cl_5HR-PIG1_35S-P | GCTCTTTAAACTATTATCATTTTTCTTATCAATCAAATAGAATACAGCGGCCGCctgcaggtccccagattagc |
|  |  |  | 3Cl_35S-P | TCTAGAtgttctctccaaatgaaatgaacttcc |
|  |  | 2027_pluc-nosp_35st | 5Cl_Myc_35S-T | GAACAAAAACTCATCTCAGAAGAGGATCTGTGACTCGAGgtccgcaaaaatcaccagtc |
|  |  |  | 3Cl_5HR-PIG1_35S-T | CCCTATGAATTATTAATATTTTAATTTGGTTATTCATGTTTTGCGGCCGCgtcactggattttggttttagg |
|  |  | pESPB001 | 5Cl_ 35S-P_ScFPR1 | GGAAGTTCATTTCATTTGGAGAGAACATCTAGAatgtctgaagtaattgaaggtaacgtc |
|  |  |  | 3Cl_Myc_ScFPR1 | CAGATCCTCTTCTGAGATGAGTTTTTGTTCgttgaccttcaacaattcgacgtc |
| DNA construct for the targeted integration of *PpFKBP12-C26Y* fused to *cMyc* | pESPB039 | Bel5-PIG1b-Act5 | 3Cl_5HR-PIG1 | TGTATTCTATTTGATTGATAAGAAAAATGATAATAGTTTAAAGAGC |
|  |  |  | 5Cl_5HR-PIG1 | AAAACATGAATAACCAAATTAAAATATTAATAATTCATAGGG |
|  |  | mAV4-hpt | 5Cl_5HR-PIG1_35S-P | GCTCTTTAAACTATTATCATTTTTCTTATCAATCAAATAGAATACAGCGGCCGCctgcaggtccccagattagc |
|  |  |  | 3Cl_35S-P | TCTAGAtgttctctccaaatgaaatgaacttcc |
|  |  | 2027_pluc-nosp_35st | 5Cl_Myc_35S-T | GAACAAAAACTCATCTCAGAAGAGGATCTGTGACTCGAGgtccgcaaaaatcaccagtc |
|  |  |  | 3Cl_5HR-PIG1_35S-T | CCCTATGAATTATTAATATTTTAATTTGGTTATTCATGTTTTGCGGCCGCgtcactggattttggttttagg |
|  |  | pDONR223-HsFKBP1A/HsFKBP12 SpectR | 5Cl_ 35S-P_HsFKBP1A | GGAAGTTCATTTCATTTGGAGAGAACATCTAGAatgggagtgcaggtggaaac |
|  |  |  | 3Cl_Myc_HsFKBP1A | CAGATCCTCTTCTGAGATGAGTTTTTGTTCttccagttttagaagctccacatc |
| PCR to validate correct integration at the PIG1 locus of the linear DNA cassette |  | Physcomitrella gDNA | 5_Up_173long_PIG1-5 | TTGTTGCAACATTTGGAGTTGGC |
|  |  |  | 3_103_35S-T(inv) | GGTTTCGCTCATGTGTTGAGC |
|  |  |  | 5_52_35S-P(inv) | CTGCGTAAGCCTCTCTAACCATC |
|  |  |  | 3_Down_81_PIG1-3 | CCAATCTGGGAATAGCTTGTTATTGTC |
| Transgene copy number determination via qPCR |  | Physcomitrella gDNA | 5-qPCR-PIG1-5HR-a | TTTCTATGCACGGATAGCAAC |
|  |  |  | 3-qPCR-PIG1-5HR-a | ACACCACACCCATCTATAGC |
|  |  |  | 5-qPCR-PIG1-3HR-b | AGACGGTTGATAGCTTGGC |
|  |  |  | 3-qPCR-PIG1-3HR-b | GCATGCTATGGACCCAAATTG |
|  |  |  | 5-qPCR-35Sp(mAV)-c | AAGGGTCTTGCGAAGGATAG |
|  |  |  | 3-qPCR-35Sp(mAV)-c | CGTTCCAACCACGTCTTC |

**Supplementary Table S3**

**Primers used to generate the estradiol-inducible *lst8*, *tor*, and *raptors* RNA interference (RNAi) systems destined for a targeted integration at the PIG1 locus in the Physcomitrella genome.**

| **Purpose** | **Targeted region within the transcript** | **Obtained vector name** | **DNA template used for PCR** | **Primer name** | **Primer sequence** |
| --- | --- | --- | --- | --- | --- |
| Estradiol-inducible *lst8* RNAi system #1 | Nucleotides 213-712 in the CDS | pESPB061 | pESPB020 | 5Cl_pENTR-PpLST8_RNAi_1st | CACCGTTGACTTATGAATCGCATATGAAC |
|  |  |  |  | 3Cl_pENTR-PpLST8_RNAi_1st | TGTGATCAGAGGACGTTGTAG |
| Estradiol-inducible *lst8* RNAi system #2 | 435 nucleotides in the 5’UTR prior to start codon | pESPB063 | Physcomitrella cDNA | 5Cl_pENTR-PpLST8_RNAi_2nd | GCGGCCGCTGCCCCTGTACAGCAAAG |
|  |  |  |  | 3Cl_pENTR-PpLST8_RNAi_2nd | GGCGCGCCCGTTTCTGACACTCACTTGC |
| Estradiol-inducible *tor* RNAi system #1 | Nucleotides 561-1060 in the CDS | pESPB065 | pESPB022 | 5Cl_pENTR-PpTOR_RNAi_1st | TGTACAAAAAAGCAGGCTCCGCGGCCGCAAAAATGCAAGTCCGAG |
|  |  |  |  | 3Cl_pENTR-PpTOR_RNAi_1st | TTGTACAAGAAAGCTGGGTCGGCGCGCCGATTTATCGCATCCTTC |
| Estradiol-inducible *tor* RNAi system #2 | Nucleotides 2496-3005 in the CDS | pESPB067 | pESPB022 | 5Cl_pENTR-PpTOR_RNAi_2nd | TGTACAAAAAAGCAGGCTCCGCGGCCGCTGTCGAGCTTCTTCAATCTG |
|  |  |  |  | 3Cl_pENTR-PpTOR_RNAi_2nd | TTGTACAAGAAAGCTGGGTCGGCGCGCCAGAACAGTAACACAAAGGCG |
| Estradiol-inducible *raptors* RNAi system #1 | Nucleotides 1656-2166 in the CDS of *PpRAPTOR1* | pESPB069 | pESPB024 | 5Cl_pENTR-PpRAPTORs_RNAi_1st | TGTACAAAAAAGCAGGCTCCGCGGCCGCGTGTCTGAACCTGGAGTTG |
|  |  |  |  | 3Cl_pENTR-PpRAPTORs_RNAi_1st | TTGTACAAGAAAGCTGGGTCGGCGCGCCACCGCTCGCAGAATGCATTC |
| Estradiol-inducible *raptors* RNAi system #2 | Nucleotides 3662-4103 in the CDS of *PpRAPTOR2* | pESPB071 | pESPB026 | 5Cl_pENTR-PpRAPTORs_RNAi_2nd | TGTACAAAAAAGCAGGCTCCGCGGCCGCTGCCAGAATCTACATTTTTCAAGTGG |
|  |  |  |  | 3Cl_pENTR-PpRAPTORs_RNAi_2nd | TTGTACAAGAAAGCTGGGTCGGCGCGCCTCCTTCTGTGTATAGTCTC |

**Supplementary Table S4**

**International Moss Stock Center (IMSC, www.mossstock-center.org) accession numbers of the lines used in this study.**

| **Code** | **IMSC accession #** | **Genotype** |
| --- | --- | --- |
| Wild type | 41269 |  |
| ESPB001 | 40976 | *PIG1bR-CaMV 35Sp-PpFKBP12-cMyc-CaMV 35St-PIG1bL* |
| ESPB002 | 40977 | *PIG1bR-CaMV 35Sp-PpFKBP12-cMyc-CaMV 35St-PIG1bL* |
| ESPB003 | 40978 | *PIG1bR-CaMV 35Sp-PpFKBP12-cMyc-CaMV 35St-PIG1bL* |
| ESPB004 | 40979 | *PIG1bR-CaMV 35Sp-PpFKBP12-cMyc-CaMV 35St-PIG1bL* |
| ESPB006 | 40980 | *PIG1bR-CaMV 35Sp-PpFKBP12^C26Y^-cMyc-CaMV 35St-PIG1bL* |
| ESPB007 | 40981 | *PIG1bR-CaMV 35Sp-PpFKBP12^C26Y^-cMyc-CaMV 35St-PIG1bL* |
| ESPB010 | 40982 | *PIG1bR-CaMV 35Sp-ScFKBP12-cMyc-CaMV 35St-PIG1bL* |
| ESPB011 | 40983 | *PIG1bR-CaMV 35Sp-ScFKBP12-cMyc-CaMV 35St-PIG1bL* |
| ESPB012 | 40984 | *PIG1bR-CaMV 35Sp-ScFKBP12-cMyc-CaMV 35St-PIG1bL* |
| ESPB014 | 40985 | *PIG1bR-CaMV 35Sp-HsFKBP12-cMyc-CaMV 35St-PIG1bL* |
| ESPB015 | 40986 | *PIG1bR-CaMV 35Sp-HsFKBP12-cMyc-CaMV 35St-PIG1bL* |
| ESPB016 | 40987 | *PIG1bR-CaMV 35Sp-HsFKBP12-cMyc-CaMV 35St-PIG1bL* |
| ESPB017 | 40988 | *PIG1bR-CaMV 35Sp-PpFKBP12-cMyc-CaMV 35St-PIG1bL* |
| ESPB018 | 40989 | *PIG1bR-CaMV 35Sp-PpFKBP12-cMyc-CaMV 35St-PIG1bL* |
| ESPB020 | 40990 | *Pplst8-RNAi-61’-40* |
| ESPB024 | 40991 | *Pplst8-RNAi-63’-38* |
| ESPB027 | 40992 | *Pptor-RNAi-65’-27* |
| ESPB032 | 40993 | *Pptor-RNAi-67’-17* |
| ESPB035 | 40994 | *Ppraptors-RNAi-69’-14* |
| ESPB040 | 40995 | *Ppraptors-RNAi-71’-25* |
